# Drug dependence is not addiction—and it matters

**DOI:** 10.1080/07853890.2021.1995623

**Published:** 2021-11-09

**Authors:** Maia Szalavitz, Khary K. Rigg, Sarah E. Wakeman

**Affiliations:** a Department of Mental Health Law & Policy, University of South Florida, Tampa, FL, USA; b Department of Medicine, Harvard Medical School, Boston, MA, USA

**Keywords:** Drug addiction, ICD, DSM, opioid use disorder, overdose crisis, physiological dependence

## Abstract

Accurately identifying persons with addiction is critically important for effectively targeting treatment and harm reduction interventions. Misdiagnosis of addictive disorders can lead to a cascade of negative outcomes, including stigma, discontinuation of needed medications, undue scrutiny of both patients and physicians, and even criminal consequences. A recent study raises significant concerns about the accuracy of diagnosis code data, likely rooted in confusingly-worded International Classification of Diseases (ICD)-9 and ICD-10 codes and a general misunderstanding of the difference between addiction and physiologic dependence. It is hardly surprising that physicians frequently mislabel patients when the ICD terms used to code for addiction are themselves misleading. ICD codes have not been updated to reflect current understanding of addiction, unlike those in the DSM-5. To explore this issue further, this commentary briefly discusses new information regarding coding data inaccuracies, how coding inaccuracies can lead to misdiagnosis, and the dangers of conflating “addiction” with “dependence.” The commentary concludes with a call for the ICD to update their codes to reflect current understanding of addiction.

**Key messages**
- It is not surprising that physicians frequently conflate patients with “addiction” and “dependence” when the ICD terms used to code for addiction are themselves misleading.
- ICD codes have not been updated to reflect what we know about the nature of addiction, unlike those in the DSM-5.
- This commentary calls for the ICD to update their codes to reflect current understanding of addiction.

Amidst the worsening polysubstance overdose crisis driven by illicitly-manufactured fentanyl, accurately identifying opioid use disorder is crucial to target effective treatment and harm reduction efforts. Frequently, payers, health care providers, and even epidemiologists utilize claims data based on diagnosis code data to guide policy and treatment. However, a recent study [1] raises significant concerns about the accuracy of these diagnostic data. The problem, we argue, is caused by confusingly-worded International Classification of Diseases (ICD)-9 and ICD-10 codes, which reflect a general misunderstanding of the difference between addiction and physiologic dependence.

Lagisetty and colleagues quantified the degree to which coding data are inaccurate in their recent paper [1]. Examining the medical records of 520 American Veterans who had been assigned ICD-9 or ICD-10 codes for “opioid use,” “opioid abuse” or “opioid dependence,” the authors found that only 57.7% showed clear signs of what the Diagnostic and Statistical Manual of Mental Disorders-5^th^ edition (DSM-5) defines as “opioid use disorder.” In fact, nearly one fifth had no indication of having any addiction issues: they were simply taking long term opioid therapy for chronic pain, which should not warrant a diagnosis at all.

In the United States, misdiagnoses of addictive disorders can lead to a cascade of negative outcomes, including stigma, discontinuation of needed medications, undue scrutiny of both patients and physicians, and even criminal consequences [2]. Misdiagnosis might also result in treatment that is inappropriate or harmful to a patient [3]. Additionally, incorrect diagnosis of addiction can threaten not just patients’ health and ability to function, but their lives. Studies have found that involuntary cessation of opioid pain treatment is associated with triple the risk of overdose death [4], as well as increased risk of suicidal thoughts and behaviour [5].

It is hardly surprising that physicians frequently mislabel patients when the ICD terms used to code for addiction are themselves misleading. ICD codes have not been updated to reflect current understanding of addiction, unlike those in the DSM-5. American experts recognized the problem as far back as 2006, when Dr. Nora Volkow, Director of the National Institute on Drug Abuse and her colleagues published an article in the *American Journal of Psychiatry* arguing that using the term “substance dependence” to mean addiction in the American Psychiatric Association’s DSM-III-R had been what they called “a serious mistake” [6].

As they put it:

The term “dependence” has traditionally been used to describe “physical dependence,” which refers to the adaptations that result in withdrawal symptoms when drugs, such as alcohol and heroin, are discontinued. Physical dependence is also observed with certain psychoactive medications, such as antidepressants and beta-blockers. However, the adaptations associated with drug withdrawal are distinct from the adaptations that result in addiction, which refers to the loss of control over the intense urges to take the drug even at the expense of adverse consequences (764) [6].

They go on to describe how physical dependence is an ordinary biological consequence of taking certain medications for weeks or years— while addiction is continued drug use that persists in the face of negative experience. This difference is reflected in the brain: brain adaptations, such as epigenetic changes can be seen in people with addiction many years after the alterations associated with and symptoms of withdrawal have resolved. Moreover, people can suffer withdrawal without having addiction and have addiction without suffering withdrawal. Indeed, nearly everyone who takes opioids for months or more will develop dependence, but only around eight percent or fewer of patients on chronic opioid therapy for pain will develop addiction [7].

Failing to distinguish between addiction and dependence, the authors argued, has:

…resulted in confusion among clinicians regarding the difference between “dependence” in a DSM (IV) sense, which is really “addiction,” and “dependence” as a normal physiological adaptation to repeated dosing of a medication. The result is that clinicians who see evidence of tolerance and withdrawal symptoms assume that this means addiction, and patients requiring additional pain medication are made to suffer. Similarly, pain patients in need of opioid medications may forgo proper treatment because of the fear of dependence, which is self-limiting by equating it with addiction (764–765) [6].

Such confusion can also contribute to a reluctance among prescribers to treat pain conditions among individuals on opioid agonist treatment.

Additional training in assessment and diagnosis for physician trainees at the medical school level is also needed. Most medical schools only devote a few hours over four years to teaching addiction medicine, a mere fraction of the time devoted to other chronic diseases encountered in general practice [8]. As a result, many physicians are ill-equipped to differentiate addiction from dependence due to a lack of expertise. Other professionals who diagnose addiction (e.g. social workers, physician assistants, nurse-practitioners, addiction counselors) also need better education about these distinctions.

Fortunately, Volkow and her colleagues’ argument carried the day with the American Psychiatric Association’s DSM-5 committee in 2013. Now, the diagnostic term for addiction in that manual is “opioid use disorder, moderate to severe.” Importantly, the DSM-5 committee also chose to remove the diagnosis of “opioid abuse,” and replace it with “opioid use disorder, mild.” This matters not just for diagnostic accuracy, but also because research has shown that the term “abuse” significantly increases punitive treatment of patients by clinicians [9].

But the ICD has yet to catch up and since American billing systems and other records often rely on ICD, this conflation continues to cause problems both in the United States and rest of the world. There is no justification for keeping this misleading term in light of what we now know about the nature of addiction. For one, depending on a substance to avoid physical withdrawal symptoms is neither necessary nor sufficient to define addiction. Many drugs cause dependence but not addiction, for example, paroxetine [10] and clonidine [11]. But when people withdraw from these medications, they do not crave them and once successfully tapered, they do not have recurrent use. In contrast, craving and recurrent use are common symptoms of addiction, particularly during early stages of recovery.

In addition, addiction can occur without physical dependence: people do not suffer visible physical withdrawal symptoms (e.g. vomiting, diarrhoea, sweating) when desisting from cocaine like they do with heroin or alcohol, but they typically have severe cravings and may even return to using [12]. Moreover, depending on something to function, in and of itself, is not pathological. Many people require a daily medication to keep a chronic condition in remission, for example insulin, blood thinners, anti-retroviral therapy, and anti-hypertensives. Dependence becomes a problem when people persist in using a substance despite its use causing harm or when its risk outweighs the benefits: in other words, when it is not just dependence, but addiction— or when severe withdrawal symptoms make cessation of a nonbeneficial medication difficult

There is also another critical reason for ICD to abandon the term “dependence” as the diagnostic label for addiction. That is, the stigma it places on medication treatment for addiction with agonists like buprenorphine and methadone. These are the only two treatments we have that are proven to cut the death rate from opioid use disorder by 50% or more [13]. However, if dependence and addiction are the same thing, then people taking methadone or buprenorphine cannot be seen as “in recovery” or “in remission”—even if they have gone from being unemployed, destitute and estranged from friends/family to becoming gainfully employed, healthy and socially connected. They are, after all, still physically dependent and would suffer withdrawal if their medication was withheld. Further, the idea that people on medication treatment are “not really in recovery” leads many to avoid the only proven lifesaving treatments we have—and has actually led to overdoses when people stop using medications to please family members or support groups that subscribe to this notion [14].

This conflation of addiction with dependence, which stigmatizes effective medication treatment for opioid use disorder, is even enshrined in law. The federal language around child abuse reporting for “substance affected” or “substance exposed” newborns has been interpreted by many states to mean that babies born to people treated with methadone or buprenorphine must be reported to child welfare authorities due to concern about abuse or neglect. This can lead to traumatic family surveillance and even separation, not surprisingly disproportionately impacting Black, Latinx, and Native American families because of racist implementation in these reporting practices.

A similar issue hurts people with chronic pain on long-term opioid therapy: if addiction is confused with dependence, they, too, are mischaracterized as having addiction. Consequently, their doctors can face legal jeopardy because maintaining someone with addiction for “comfort” is not considered a legitimate medical purpose under American law, except in strictly limited circumstances and only if the medication being used is methadone [14] or buprenorphine [15]. Although there have been many cases of inappropriate use of prescription opioids for chronic pain, it is also clear that some patients do benefit [16]. It has been nearly a decade since the American Psychiatric Association recognized that the DSM needed to stop conflating addiction and dependence. The time has come for the ICD to follow suit.

## Data Availability

Data sharing is not applicable to this article as no new data were created or analysed in this study.

## References

[1] Lagisetty P, Garpestad C, Larkin A, et al. Identifying individuals with opioid use disorder: validity of international classification of diseases diagnostic codes for opioid use, dependence and abuse. Drug Alcohol Depend. 2021;221:108583. 33662670 10.1016/j.drugalcdep.2021.108583 PMC8409339

[2] Stone EM, Kennedy-Hendricks A, Barry CL, et al. The role of stigma in U.S. primary care physicians’ treatment of opioid use disorder. Drug Alcohol Depend. 2021;221:108627. 33621805 10.1016/j.drugalcdep.2021.108627 PMC8026666

[3] Sargent WC, Shaffer HJ, Lawford C. Two cases of misdiagnosis: some essentials of intake. J Subst Abuse Treat. 1986;3(2):69–75. 3761410 10.1016/0740-5472(86)90055-3

[4] James JR, Scott JM, Klein JW, et al. Mortality after discontinuation of primary care–based chronic opioid therapy for pain: a retrospective cohort study. J Gen Intern Med. 2019;34(12):2749–2755. 31468341 10.1007/s11606-019-05301-2 PMC6854174

[5] Demidenko MI, Dobscha SK, Morasco BJ, et al. Suicidal ideation and suicidal self-directed violence following clinician-initiated prescription opioid discontinuation among long-term opioid users. Gen Hosp Psychiatry. 2017;47:29–35. 28807135 10.1016/j.genhosppsych.2017.04.011

[6] O’Brien CP, Volkow N, Li T-K. What’s in a word? Addiction versus dependence in DSM-V. AJP. 2006;163(5):764–765. 10.1176/ajp.2006.163.5.764 16648309

[7] Volkow ND, McLellan AT. Opioid abuse in chronic pain–misconceptions and mitigation strategies. N Engl J Med. 2016;374(13):1253–1263. 27028915 10.1056/NEJMra1507771

[8] Tetrault JM, Petrakis IL. Partnering with psychiatry to close the education gap: an approach to the addiction epidemic. J Gen Intern Med. 2017;32(12):1387–1389. 28766126 10.1007/s11606-017-4140-9 PMC5698217

[9] Kelly JF, Dow SJ, Westerhoff C. Does our choice of substance-related terms influence perceptions of treatment need? An empirical investigation with two commonly used terms. J Drug Issues. 2010;40(4):805–818.

[10] Fava GA, Gatti A, Belaise C, et al. Withdrawal symptoms after selective serotonin reuptake inhibitor discontinuation: a systematic review. Psychother Psychosom. 2015;84(2):72–81. 25721705 10.1159/000370338

[11] Houston MC. Abrupt cessation of treatment in hypertension: consideration of clinical features, mechanisms, prevention and management of the discontinuation syndrome. Am Heart J. 1981;102(3 Pt 1):415–430. 6115570 10.1016/0002-8703(81)90317-3

[12] National Library of Medicine Medline Plus Medical Encyclopedia. Cocaine withdrawal [cited 2019 January 12]. Available from: https://medlineplus.gov/ency/article/000947.htm

[13] Pierce M, Bird SM, Hickman M, et al. Impact of treatment for opioid dependence on fatal drug-related poisoning: a national cohort study in England. Addiction. 2016;111(2):298–308. 26452239 10.1111/add.13193 PMC4950033

[14] Wakeman S, Szalavitz M. Why taking drugs to treat addiction doesn’t mean you’re ‘still addicted’ [cited 2017 May 18]. Available from: https://www.statnews.com/2017/05/18/opioids-medication-assisted-therapy/

[15] Fiscella K, Wakeman SE, Beletsky L. Buprenorphine deregulation and mainstreaming treatment for opioid use disorder: X the X waiver. JAMA Psychiatry. 2019;76(3):229–230. 30586140 10.1001/jamapsychiatry.2018.3685

[16] Noble M, Treadwell JR, Tregear SJ, et al. Long‐term opioid management for chronic noncancer pain. Cochrane Database Syst Rev. 2010;(1):CD006605. 20091598 10.1002/14651858.CD006605.pub2 PMC6494200

